# Political Medicine; the Substance of a Discourse on Medicine, Considered in Its Relations to Government and Legislation

**Published:** 1839-10

**Authors:** 


Art. XVIII.—*Political Medicine; being the substance of a Discourse lately delivered before the Royal College of Surgeons in Ireland, on Medicine, considered in its relations to Government and Legislation.* By H. Maunsell, m.d., one of the Professors in the Royal College of Surgeons in Ireland.—*Dublin*, 1839. 8vo, pp. 45.

We recommend this pamphlet to all our readers, as treating admirably of a subject of the very first importance, but one which has been neglected, in these kingdoms, to an extent which would be truly astonishing, were it not well known to be one of the common results of a popular government and free institutions, that the people are, for the most part, left to regulate their own affairs in the way they prefer. For our own parts, although we are as jealous of our political freedom as any one, and would much rather bear a hundredfold more of the social and economical evils, which are its legitimate offspring, than be tyrannised into better manners and healthier habits by the most benevolent of despotisms—still we do think that the government of this country might interfere more than it does in not a few matters of domestic and social life, with infinite advantage to the community, and without trenching, in any degree, on the wholesome liberty of the subject. And in no part of our social economy would such an interference be more justifiable and more beneficial than in regulating those branches of medicine which relate to the public health, or the means of preventing diseases, and of mitigating their ravages when they prevail. In this respect Great Britain is far behind almost all other European countries. The object of

Dr. Maunsell's pamphlet is to call attention to this department of medicine (termed by him *political medicine*), and of which the surpassing importance is well and clearly illustrated in its pages. In relation to his subject the author rapidly reviews the questions of vaccination, the health of the army and navy, the health of prisons, the quarantine system, epidemic and endemic diseases, unwholesome trades, lunatics and lunatic asylums, medical attendance on the poor, colonization and emigration, &c. It is quite clear that, for the due regulation of these and other similar matters, of the highest moment to the welfare of the community, there must be a general reform in the actual constitution of the medical profession generally. One necessary result of this would be the formation of a Board of Health, constructed on liberal and sound principles, to be the organ of communication with the government on all questions of medical police. We are happy to think that things are progressively, though slowly, ripening towards so desirable a consummation; and as pointing out some of the grievous defects and evils of our present system, and a few of the benefits to be expected from a better, Dr. Maunsell's little work will be highly serviceable to the great cause of medical reform; we therefore cordially recommend it to the notice of our readers.

